# UVA, UVB and UVC Light Enhances the Biosynthesis of Phenolic Antioxidants in Fresh-Cut Carrot through a Synergistic Effect with Wounding

**DOI:** 10.3390/molecules22040668

**Published:** 2017-04-24

**Authors:** Bernadeth B. Surjadinata, Daniel A. Jacobo-Velázquez, Luis Cisneros-Zevallos

**Affiliations:** 1 Department of Horticultural Sciences, Texas A&M University, College Station, TX 778432-133, USA; Bernadeth_Surjadinata@bayvalleyfoods.com; 2 Tecnológico de Monterrey, Escuela de Ingeniería y Ciencias, Centro de Biotecnología FEMSA, Av. Eugenio Garza Sada 2501 Sur, C.P. 64849 Monterrey, N.L., Mexico; djacobov@itesm.mx

**Keywords:** wounding, UVA UVB UVC radiations, phenolics, carrots, ROS mediated mechanism

## Abstract

Previously, we found that phenolic content and antioxidant capacity (AOX) in carrots increased with wounding intensity. It was also reported that UV radiation may trigger the phenylpropanoid metabolism in plant tissues. Here, we determined the combined effect of wounding intensity and UV radiation on phenolic compounds, AOX, and the phenylalanine ammonia-lyase (PAL) activity of carrots. Accordingly, phenolic content, AOX, and PAL activity increased in cut carrots with the duration of UVC radiation, whereas whole carrots showed no increase. Carrot pies showed a higher increase compared to slices and shreds. Phenolics, AOX, and PAL activity also increased in cut carrots exposed to UVA or UVB. The major phenolics were chlorogenic acid and its isomers, ferulic acid, and isocoumarin. The type of UV radiation affected phenolic profiles. Chlorogenic acid was induced by all UV radiations but mostly by UVB and UVC, ferulic acid was induced by all UV lights to comparable levels, while isocoumarin and 4,5-diCQA was induced mainly by UVB and UVC compared to UVA. In general, total phenolics correlated linearly with AOX for all treatments. A reactive oxygen species (ROS) mediated hypothetical mechanism explaining the synergistic effect of wounding and different UV radiation stresses on phenolics accumulation in plants is herein proposed.

## 1. Introduction

It has been previously reported that the phenolic content and antioxidant capacity (AOX) in carrots increases with wounding intensity [1,2,3]. These changes in phenolic content are triggered by ROS and ethylene, which are signaling molecules that activate the phenylpropanoid pathway in wounded-plants [2,4]. Likewise, previous reports have demonstrated that when wounding stress is applied in combination with other stresses such as exogenous phytohormones [3], hyperoxia [2], glyphosate [5] and water-loss [6], the accumulation of phenolic compounds in carrots is affected.

Several studies have reported that UV radiation may trigger the phenylpropanoid metabolism in plant tissues [7,8,9,10,11,12,13,14,15]. UV radiation can be divided into three parts: UVA (320–400 nm), UVB (280–320 nm), and UVC (200–280 nm). UVA represents about 6% of the total solar radiation and is the least harmful part of UV radiation. UVB can cause a variety of damaging effects in plants and it represents approximately 1.5% of the total spectrum. UVC is very hazardous to organisms; however, the stratospheric ozone layer filters out most of this UV radiation [16].

It is recognized that there are both direct and indirect effects of UV radiation on plants, which include changes in photosynthesis, cell division, change in DNA, and other processes related to growth and development. These effects are observed only after a few hours or days of exposure. Besides these damaging effects, plants also have “adaptive” responses, such as activation of different defensive mechanisms as protection against UV radiation. The most studied mechanism is the production of flavonoids, especially anthocyanins to screen out UVB [9].

Plants must be able to deal with potentially harmful stress conditions that are almost constantly present in their environment. Since they cannot move, they have limited capability to avoid these stresses. Each cell must have mechanisms that allow any signals to be detected and acted upon to give rise to particular responses.

Plants exhibit physiological responses to protect them from damaging UV radiation. These responses include the synthesis of flavonoids, hydroxycinnamic acids and their related compounds. These responses are likely to involve specific UV photoreceptors and signal transduction processes, which lead to the regulation of gene transcription [9]. Accumulation of these protective compounds occurs primarily in epidermal layers [9,17]. Flavonoids have λ_max_ at 270 and 345 nm, whereas hydroxycinnamic acids have λ_max_ at 320 nm, so both classes may protect against damaging UV radiation. Alternatively, UV radiation may interact with other atoms or molecules in the cell, particularly with water, which then produces free radicals [18]. These radicals can diffuse far enough to reach and harm different components of the cell. This effect is very significant in plants since the cytoplasm of the plant cell contains about 80% water [18]. Plant cells may also respond to these reactive oxygen species (ROS) by triggering the phenylpropanoid metabolism [2,4].

The present project’s objective was to investigate the effects of UVA, UVB, and UVC radiation alone and in combination with wounding stress on the phenylpropanoid metabolism of carrots by measuring the total and individual phenolic compounds, the antioxidant capacity (AOX), and the phenylalanine ammonia-lyase (PAL) activity through time. The results presented herein are based on the dissertation work of Surjadinata [19], which were confirmed in further studies, where the effects of UVB [14,15] and UVC [13] on the AOX and phenolic content of carrots were determined. To the best of our knowledge, this is the first literature report evaluating the different types of UV light on the phenylpropanoid metabolism of carrots and proposing a mode of action.

## 2. Results and Discussion

### 2.1. Effect of UVC and Different Wounding Intensity (A/W) on Total Phenolic, PAL Activity and AOX during Storage

**Figure 1:**
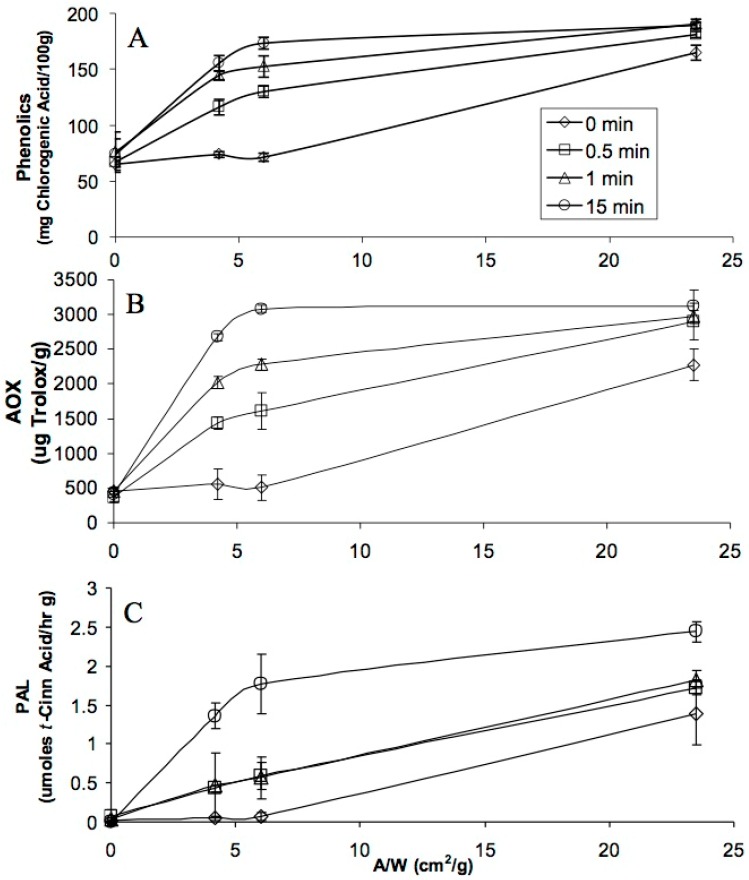
Total phenolic content (**A**); antioxidant capacity (**B**); and phenylalanine ammonia-lyase (PAL) activity (**C**) of carrots cuts (A/W) including whole, slices, pies and shreds radiated with different doses of UVC (11.8 Watts/m^2^ for 0, 0.5, 1 and 15 min). Measurements were taken after 4 d of storage at 15 °C. All quantifications were on fresh weight basis. Vertical bars represent standard deviations (*n* = 5).

The total phenolics, AOX and PAL activity of carrots was affected by wounding stress alone or in combination with UVC light (Figure 1). There was a significant increase (*p* < 0.05) in phenolic content compared to controls (non-wounded) and wounded tissues radiated with different UVC exposure times. The interaction between wounding and UVC stresses was synergistic since non-wounded tissue was basically not affected by the UVC treatment. Compared to the non-radiated treatment, total phenolics in cut tissue increased 111, 143, and 15%, for slices (A/W = 4.2 cm^2^/g), pies (A/W = 6.0 cm^2^/g), and shreds (A/W = 23.5 cm^2^/g), respectively, after exposure to UVC for 15 min (Figure 1A). In general, for each wounding intensity, there was a dose response when UVC was applied. The dose response decreased as follows: pies > slices > shreds > non-wounded. Interestingly, although the highest PAL activity was detected in carrot tissue treated with the highest wounding intensity and UVC dose, this tissue did not show the highest accumulation of phenolic compounds. This could be related either to the availability of the carbon source (sugars and amino acids) needed for phenolic biosynthesis [20] or to the conversion of phenolics into lignin [6,21].

After 15 min of UVC exposure, AOX increased 384, 506, and 37% for slices, pies, and shreds, respectively (Figure 1B). The trend followed by the increase in AOX was similar to that of phenolics for each wounding intensity. The oxidative burst caused by UVC exposure must be counteracted by antioxidants and protective pigments, such as flavonoids and carotenoids, to prevent cell damage [22]. This could partially explain the increase in antioxidant capacity when the cut carrot tissues were radiated with UVC. ROS induction is known to be one of the early effects of exposure to UV lights. Vicente et al. [23] also found an increase in the antioxidant capacity of UVC treated peppers stored at 10 °C for a period of 18 days.

PAL activity increased almost 30, 27, and 0.75 folds for slices, pies, and shreds respectively, after the cut tissues were radiated for 15 min of UVC and stored at 15 °C for four days (Figure 1C). It is well established that light activates the expression of genes encoding PAL, chalcone synthase (CHS), and several other phenylpropanoid enzymes [9,24,25].

Figure 1 also illustrates that non-wounded carrot tissues (A/W = 0 cm^2^/g) radiated with UVC did not show a significant increase (*p* > 0.05) in the total phenolics, AOX and PAL activity. It seems that for UVC to affect carrot tissue, there is a need of an initial wounding stress to take place. It has been shown that plants make decisions and prioritize for certain stress responses while down-regulating another response [26,27,28]. However, in the present study, a simple test performed by adding a thin layer of carrot peel onto the UVC detector showed negligible readings, indicating that UV light does not penetrate the carrot skin or cuticle.

### 2.2. Wounded Carrot Exposed to UVA, UVB and UVC Lights

**Figure 2:**
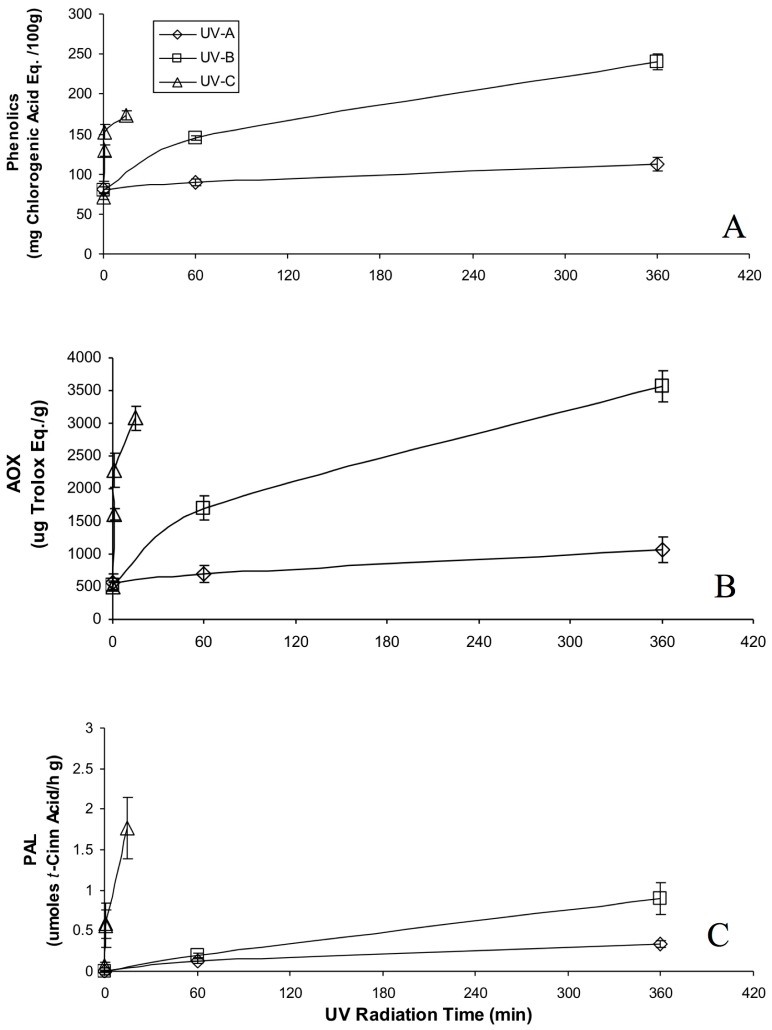
Total phenolic content (**A**); antioxidant capacity (**B**); and PAL activity (**C**) of carrot pies radiated with different UV lights (light intensities of 11.8, 10.4 and 12.7 W/m^2^ for UVC, UVB and UVA, respectively). Measurements were taken after 4 d of storage at 15 °C. All quantifications were on fresh weight basis. Vertical bars represent standard deviations (*n* = 5).

According to the results obtained on the effect of UVC light in combination with different wounding intensities on the phenylpropanoid metabolism of carrots, carrot pies were selected to further evaluate the effect of different UV radiations (UVA, UVB and UVC) on total phenolics, phenolic profile, AOX and PAL activity (Figure 2).

After 360 min of UV radiation, total phenolic content of carrot pies increased 1 and 3 folds, AOX capacity increased 2 and 7 folds, and PAL activity increased 34 and 90 folds, for UVA and UVB, respectively (Figure 2). The dose response curve for UVA, UVB, and UVC were different. Tissue response to the type of UV decreased as follows: UVC > UVB > UVA. Despite the intensity of all UV lights being similar (~10.4–12.7 W/m^2^), the differing exposure times provided higher energy doses for UVA and UVB compared to UVC. However, it appears that the shorter wavelength of UVC compared to UVB and UVA has a larger impact on generating the common signal that triggers the response.

**Figure 3:**
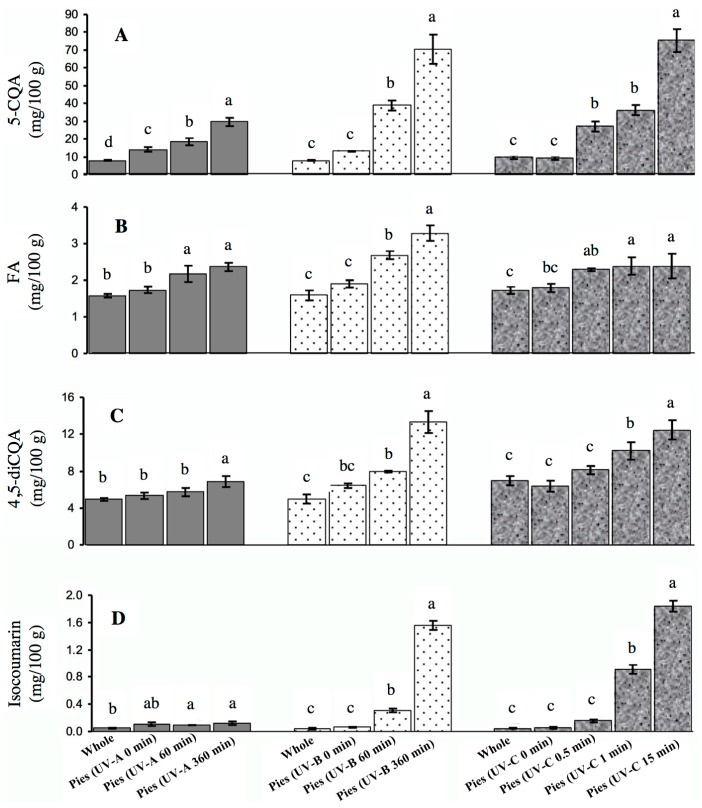
Effect of different UV radiations on individual phenolic compounds of carrot tissue; chlorogenic acid (5-CQA) (**A**); ferulic acid (FA) (**B**); 4,5-dicaffeoylquinic acid (4,5-diCQA) (**C**); and isocoumarin (**D**). Measurements were taken after 4 d of storage at 15 °C. All quantifications were on fresh weight basis. Vertical bars represent standard deviations (*n* = 5). Bars with different letters indicate statistical difference (*p* < 0.05).

The application of different UV lights to the wounded tissue affected the concentration of individual phenolic compounds (Figure 3). Individual phenolics were identified with reverse phase HPLC-DAD, by comparing their retention times and absorption spectra with reference standards or previous reports where LC-MS was used for the identification [5,6]. The major phenolic compounds affected by UV radiation in combination with wounding included chlorogenic acid (5-CQA), ferulic acid (FA), 4,5-dicaffeoylquinic acid (4,5-diCQA), and isocoumarin. Other phenolic compounds such as *p*-hydroxybenzoic acid (*p*HBA), *p*-cumaric acid derivative, 3,5-dicaffeoylquinic acid (3,5-diCQA), and an HBA derivative were also detected but in minor quantities (supplementary information, Figures S1–S3, Tables S1–S3).

In general, the individual phenolic content induced by exposure to UVB is higher than UVA and comparable to UVC. A 360 min exposure of carrot tissue to UVA or UVB, or a 15 min exposure to UVC only induced 0.12, 1.56 and 2 mg isocoumarin/100 g fresh weight tissue, respectively. It is known that isocoumarin exerts a bitter flavor in carrots, however, the concentrations detected in UV treated carrots are below the maximum level (≥20 mg/100 g of fresh weight) for bitterness detection [29]. In addition to the compounds shown in Figure 3, other phenolics including *p*HBA, 3,5-diCQA, *p*-coumaric acid derivative, and HBA derivative, were accumulated only in UV treated samples but not in non-UV radiated control carrot pies (supplementary information, Tables S1–S3). For instance, *p*HBA was detected in UVA and UVC treated pies at 0.37 and 1.16 mg/100 g, respectively. Similarly, the 3,5-diCQA was induced in UVA and UVB treatments at levels of ~4.60 mg/100 g, while *p*-coumaric acid derivative and HBA derivative were only present in UVB and UVC but not in UVA.

The specific AOX increased as UV radiation time increased (Table 1), which indicates that wounding stress combined with UV exposure induced phenolic compounds with more AOX capacity [30]. For tissue exposed to UVA, there was no significant difference (*p* > 0.05) in specific AOX at different UVA doses. However, for tissue exposed to UVB and UVC, the specific AOX increased with UV exposure time (Table 1).

Table 1 also shows the relative proportions of the three major hydroxycinnamic acids present in the wounded carrot tissue exposed to different UV doses: chlorogenic acid (5-CQA), ferulic acid (FA), and one of the chlorogenic acid isomers (4,5-diCQA). The different UV doses give different relative proportions of 5-CQA: FA: 4,5-diCQA. For example, FA and 4,5-diCQA proportions decreased with increased UV time exposure. This means that the primary compound that determined the specific phenolic antioxidant is chlorogenic acid or 5-CQA. Interestingly, when the effect of all three UV lights were combined into one figure, it can be observed that certain phenolic compounds were differentially triggered by the type of UV light. Figure 3A shows that chlorogenic acid is synthesized by all UV lights but in higher quantities by UVB and UVC, while Figure 3B shows that ferulic acid is induced to comparable levels by all UV lights. For 4,5-diCQA and isocoumarin, they are synthesized in higher quantities by UVB and UVC, compared to UVA (Figure 3C,D).

In general, results showed that exposing carrot tissue to different UV lights increases the phenolic contents. There are not many studies that have investigated the effect of UVA on the phenolic contents of fruit and vegetable tissues. There are some studies that investigated the effect of UVB on the synthesis and accumulation of anthocyanin in light-colored sweet cherry [31]. Reay and Lancaster [11] found a similar result, where anthocyanins and quercetin glycosides were induced in Gala and Royal Gala apple fruit skin after radiation with both UVB and visible lights.

Many studies have also been conducted to study the effect of UV radiations, mostly UVB, on growing plants or trees. Many of these plants contain a wide variety of phenolics, including cinnamic acids, chlorogenic acids, glycosylated flavonoids, and tannins [32]. They are present in all tissues, including leaves, stems, buds, flowers, and even roots. The existence of these secondary metabolites is fairly species-specific and plant-part specific. In addition, the relative composition may change dramatically over the development period of the plant, from seedlings to maturity [32].

Most of these phenolics are UV absorbing compounds. They play an important role in plant tolerance of UV radiation because they can reduce UV penetration into the plant tissue and they also act as antioxidants, protecting the plants from damage caused by ROS [32].

### 2.3. Potential Signaling Mechanism of UVA, UVB and UVC Radiation Stress in Carrot Tissue

The mechanisms by which plants perceive UV radiation and initiate physiological responses to it are not fully understood. Most of the previous reports have been performed using UVB radiation, since the effect of UVA is not significant in plants and most of UVC radiation is filtered out by the stratospheric ozone layer. Likewise, UVC radiation has been mostly used in harvested commodities as a tool to extend their shelf-life [33,34,35]. Therefore, there is a lack of information on the effects of UVA and UVC on plants at the cellular and molecular levels.

Jenkins et al. [7] proposed several possible mechanisms for the specific detection of UVB. The detection of signals in many cases is likely to involve specific cellular components called receptors [7]. Reception is coupled to the terminal response by signal transduction mechanisms. The signal transduction process often serves to amplify the initial signal and in some cases, may store it for certain periods of time [7].

The first proposed theory is that direct absorption of UVB by DNA in the nucleus could result in the generation of some type of signals, which stimulate the rate of transcription of several genes. The DNA molecule itself has been considered a candidate for a UVB receptor because many responses related to UVB were maximally stimulated by wavelengths between 250 and 280 nm [36]. However, the action spectra of UVB responses in plants revealed their maximal stimulation was between 290 and 310 nm [36]. Additionally, there is a lack of correlation between the increase of DNA damage and the UVB elicited changes in the transcript profile [37]. Therefore, these evidences contradict the theory that damaged DNA serves as a UVB receptor.

Another possible mechanism involves the detection of UVB by photoreceptor molecules similar to other photoreception systems in higher plants. Plants have developed a photo-sensory system that can monitor the change in their light environment. This system contains three known classes of photoreceptors: the phytochromes (PHY) for far-red and red lights, the crytochromes (CRY) for blue light, and the phototropins (PHOT) for UVA light [38]. The photoreceptor molecules for UVB and UVC are still unidentified [38]. In our situation, this proposed mechanism seems unlikely since carrot is a root. Naturally, carrot root grows underground, thus is not likely to have photoreceptors.

There are also other possible signaling molecules that are involved in the regulation of gene expression due to UV radiation. A-H-Mackerness et al. [39] studied three different signaling pathways that control UVB-induced stress on *Arabidopsis thaliana*: ROS, jasmonic acid, and ethylene. UV could be detected by the plant’s ability to generate reactive oxygen species. In this case, the increase in transcription observed after UV exposure would be oxidative stress responses rather than photo-responses to UV wavelengths. The study by Green and Fluhr [40] supports this hypothesis. They found that the UVB response was greatly reduced when antioxidants were applied to tobacco leaves. The increase in AOX capacity of carrot tissue after wounding and UV radiation stresses in the present study could be linked to this possibility of ROS as the signaling molecule. UVB exposure to *Arabidopsis* has been shown to induce activities of the antioxidant enzymes system, such as guaiacol peroxidase, ascorbate peroxidase, and peroxidase [41]. On the other hand, applications of superoxide dismutase (SOD) and catalase (CAT), which are also components of the antioxidant enzyme system, have been proven to reduce the effect of UVB on *Arabidopsis* [39].

Besides ROS, another possible pathway activated by high doses of UV is linked to wound signal transductions, which involve jasmonic acid and ethylene [4,39]. These molecules have been proposed to trigger the synthesis of phenolic antioxidants as a defense mechanism against these stresses. The role of ethylene in signal transduction pathways leading to gene expression in response to UVB has been shown by A-H-Mackerness et al. [39] in *Arabidopsis* mutant and by Wang et al. [42] in leaves of maize seedlings. However, there is also the possibility that the above mechanisms are not solitary. It is feasible that UVB regulates gene expression by combining those mechanisms. According to A-H-Mackerness et al. [39], the increase in ROS levels after exposure to UVB could lead to an increase in levels of jasmonic acid and ethylene. Overlap responses such as these have been shown to extend from the transcriptional level to the intercellular signaling pathways that regulate gene expression [28,39]. As mentioned earlier, application of SOD and CAT enzymes has been shown to reduce the effect of UVB on the gene transcripts in *Arabidopsis*. This effect of SOD and CAT is likely through their activity at the surface of the cells, since they are unlikely to penetrate the plasma membrane [39].

**Figure 4:**
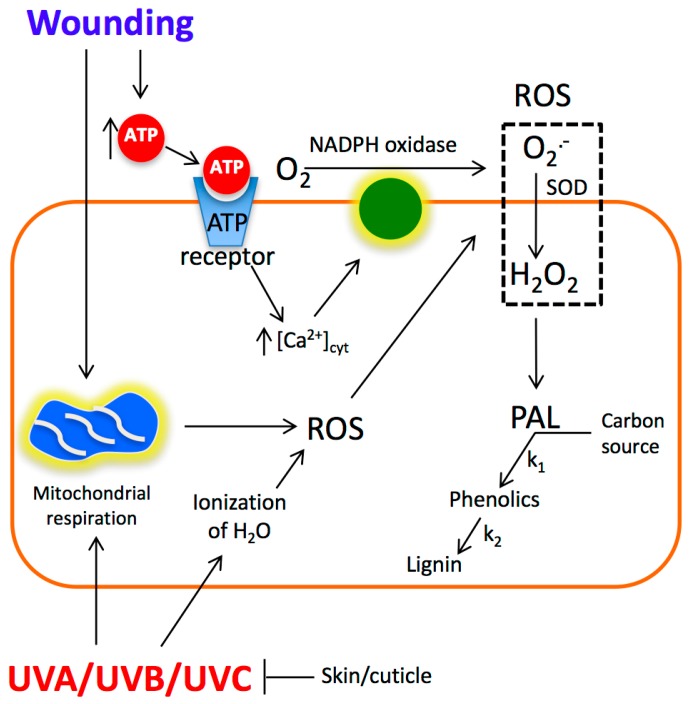
Proposed model explaining the synergistic effect of wounding and UVA, UVB and UVC radiation stresses on phenolics accumulation in carrots. UV radiation produces reactive oxygen species (ROS) as a primary signal for the activation of PAL and for the accumulation of phenolics. ROS production induced by UV is triggered by increasing mitochondrial respiration and by water ionization. The skin/cuticle of plants contains UV light-filtering compounds, thus the amount of ROS induced by UV would be tissue-dependent. When wounding is applied prior to UV radiation, the skin/cuticle would be partially removed, and thus the area for the penetration of radiation is increased, leading to higher ROS production, increased PAL activity and phenolics accumulation. On the other hand, wounding has its own mechanism of ROS production. For wounding, ATP acts as the primary signal for the production of ROS which acts like a secondary signal increasing the levels of PAL and phenolics. Abbreviations: SOD (superoxide dismutase); PAL (phenylalanine ammonia-lyase); ROS (reactive oxygen species); k_1_ (rate of phenolic biosynthesis); k_2_ (rate of phenolic utilization).

Recently, we proposed a mechanism for the wound-induced accumulation of phenolic compounds in carrots [2,4]. Likewise, we reported the mechanism of UV induced modulation of phenolics and vitamin C in acerola [43]. Based on those reports and the present study, a hypothetical model explaining the synergistic effect of wounding and UV radiation stresses on the accumulation of phenolic compounds in plants is proposed in Figure 4.

UVA, UVB and UVC radiation produces ROS as a primary signal for the activation of PAL and for the accumulation of phenolics [43]. ROS production induced by UV is triggered by increasing mitochondrial respiration and by water ionization [43]. The skin/cuticle of plants contains UV light-filtering compounds, thus the amount of ROS induced by UV would be tissue-dependent. For instance, in the specific case of acerola, UVC radiation could penetrate the tissue, increase ROS production and activate the phenylpropanoid metabolism. On the other hand, in the specific case of whole carrots, the skin/cuticle filters the light (according to measures performed in the present study with UV sensors) and thus no significant changes in the production of phenolics was observed. When wounding is applied prior to UV radiation, the skin/cuticle would be partially removed, and thus the area for the penetration of radiation is increased, leading to higher ROS production acting as primary signal for UV, increased PAL activity and phenolics accumulation. On the other hand, wounding has its own mechanism of ROS production where ATP acts as the primary wound signal while ROS works as a secondary signal [4]. Thus, when both stresses are applied simultaneously, a synergistic effect on the accumulation of phenolics is observed. The amount of phenolics accumulated will be dependent on the availability of the carbon source (sugars and aminoacids) associated to the rate (k_1_) of phenolic biosynthesis [20] and the rate of phenolic utilization (k_2_), like in the production of lignin [6,21].

## 3. Materials and Methods

### 3.1. Plant Materials and Reagents

Carrots cultivar Legend, which were grown in California (Grimmway Farms, Bakersfield, CA, USA) were examined. This cultivar is commonly used for processing by the fresh produce industry. To decrease variability, all carrots used in the experiment were approximately the same size and were inspected for any visual damage because any injury prior to processing could induce PAL activity. The selected carrots were stored in a 15 °C room until they were ready to be processed.

All chemicals and standards used: Folin-Ciocalteu reagent, sodium carbonate (Na_2_CO_3_), Trolox and 2,2-diphenyl-1-picrylhydrazyl (DPPH), polyvinylpyrrolidine (PVPP), sodium hydroxide (NaOH), boric acid, 2-mercaptoethanol, chlorogenic acid, *p*-hydroxybenzoic acid, *p*-coumaric acid, and ferulic acid were purchased from Sigma Chemical Co. (St. Louis, MO, USA). Methanol and acetonitrile were reagents of HPLC grade.

### 3.2. Wounding and UV Radiation Stresses

Carrots were processed after being washed with 100 ppm chlorinated water and dried at room temperature for 2–3 h. For the first experiment, carrots were cut into slices, pies and shreds. Intensity of wounding, A/W of 4.2, 6.0, and 23.5 cm^2^/g, respectively, were calculated based on our previous work [44]. Whole non-wounded carrots (A/W = 0 cm^2^/g) were used as the control. These carrots were then exposed to two UVC lamps (G40T-10 lamp, Sankyo Denki, Hiratsuka, Japan) for 0, 0.5, 1, and 15 min at room temperature (UV chamber size of 120 cm × 60 cm × 54 cm with reflective panels). The carrots were placed on weighing dishes on a single layer ~50 cm below the UV light. The UVC intensity was determined as 11.8 W/m^2^ using a UV521C radiometer equipped with a UVC sensor measuring in the spectral range of 220–275 nm (General Tools & Instruments, New York, NY, USA). The carrots were inverted in the middle of the radiation process to achieve optimum exposure on all sides of the tissue.

In a second experiment, carrot pies were exposed to four lamps of UVA (UVA-351 lamp, Q-Panel Lab) or UVB (UVB-313 lamp, Q-Panel Lab) for 0, 60, and 360 min at room temperature. The cut carrots were also placed on weighing dishes in a single layer and were positioned 50 cm from the UV light. The UV intensities were determined as 12.73 and 10.44 W/m^2^ for UVA and UVB, respectively, using a PMA 2200 radiometer equipped with PMA 2110 UV-A and PMA 2106 UV-B sensors (Solar Light, Glenside, PA, USA) measuring in the spectral range from 320–400 nm and 280–320 nm, respectively. The carrots were inverted in the middle of the radiation treatment for maximum UV exposure. The relative humidity of the UV chamber was kept above 90% by providing continuous humidified air-flow onto the tissue. This was done to prevent moisture loss during radiation.

After treatment, the stressed carrots were placed in 4 L closed glass jars, stored in the dark at 15 °C, and ventilated every 8–12 h to avoid CO_2_ accumulation. All treatments had five replicates of similar weight (around 150 g). Measurements were done after four d of storage.

To determine if UV light penetrates the carrot skin or cuticle, a thin layer of carrot peel (~0.9 mm) was obtained with a hand vegetable peeler. In addition, cells were scrapped off from the peel with a knife to obtain a thinner skin layer (~0.3 mm). Both skin samples were taped on top of the UV sensors showing negligible readings of UV intensities of ~0.025% and 0.05%, respectively, of the corresponding UV lights used.

### 3.3. Phenolics, AOX, and PAL Activity

All quantifications were done according to Surjadinata and Cisneros-Zevallos [1]. Total phenolics were measured using Folin-Ciocalteau reagent, assayed spectrophotometrically at 725 nm. Individual phenolics were identified with reverse phase HPLC-DAD, by comparing their retention times and absorption spectra with reference standards or previous reports [5,6].

Total phenolic content was expressed as mg chlorogenic acid equivalent/100 g fresh weight tissue. Antioxidant capacity was measured using DPPH radical protocol, measured spectrophotometrically at 515 nm and expressed as μg Trolox equivalents/g fresh weight tissue. PAL activity was assayed with spectrophotometer at 290 nm and expressed as μmoles of t-cinnamic acid/h g fresh weight tissue.

### 3.4. Statistical Analysis

Statistics analysis was done using the ANOVA procedure from the SAS Statistical Analysis System for Windows v8.1 (SAS Institute Inc., Cary, NC, USA). The treatment means were compared with Tukey’s Studentized Range test at α = 0.05.

## 4. Conclusions

Combining abiotic stresses, such as wounding and UV light radiation, increased the phenolic content, antioxidant capacity, and PAL activity of carrot tissue. The increase was more significant and rapid when the tissue was exposed to UVC for a few minutes. Exposing the tissue to UVB could also increase the phenolic content to a similar level as UVC exposure, but it takes more time to exert the response (hours, instead of minutes). As for radiation with UVA, it also induced the synthesis of phenolic content, however, this increase was not as considerable as the other two UV lights. Different wounding intensity (A/W) also affected the response of the tissue to UV radiation. The carrot pies have the highest synergistic effect of wounding and UV stresses, even though the A/W is not as high as shredded carrots. There is a maximum amount of phenolics that can be synthesized which will depend on the availability of a carbon source and/or the utilization of phenolics, like lignin production.

The main phenolic compound induced by both wounding and UVA, UVB and UVC radiation was chlorogenic acid, which is a nutraceutical that can prevent and treat diseases associated with metabolic syndrome [45]. Therefore, the application of wounding in combination with UV radiation could be used by the fresh-cut produce and food processing industries as a simple tool to increase the health-promoting properties of carrots.

## Figures and Tables

**Table 1:** Relative proportions of the three major hydroxycinnamic acids present (%) and the specific AOX ± SD (*n* = 5) of carrot pies tissue exposed to UVA, UVB and UVC lights.

	Exposure Time (h)	5-CQA %	FA %	4,5-diCQA %	Specific AOX (µg Trolox/mg Phenolic) ^1^
UVA	0	66.7	7.7	25.6	703 ± 93.87
	1	69.6	8.7	21.7	776 ± 119.74
	6	76.8	5.4	17.9	950 ± 118.15
UVB	0	60.6	9.1	30.3	658 ± 50.63
	1	79.0	4.8	16.1	1173 ± 118.18
	6	80.3	4.5	15.2	1485 ± 49.44
UVC	0	51.9	11.1	37.0	712 ± 42.85
	0.5 min	71.7	6.5	21.7	1239 ± 31.79
	1 min	75.0	4.2	20.8	1495 ± 90.72
	15 min	83.6	2.7	13.7	1776 ± 73.98

^1^ Phenolic content was quantified based on 5-CQA spectrophotometric standard curve using the Folin-Ciocalteu assay.

## Supplementary Materials

The following are available online.
