# Supplementary material for: UVA, UVB and UVC Light Enhances the Biosynthesis of Phenolic Antioxidants in Fresh-Cut Carrot through a Synergistic Effect with Wounding
Source: Molecules. 2017 Apr 24;22(4):668. doi: 10.3390/molecules22040668 (PMC6154643; doi:10.3390/molecules22040668)
Supplement: Supplementary file 1 [file molecules-22-00668-s001.pdf]

Supplementary information

UVA, UVB and UVC Light Enhances the Biosynthesis of Phenolic Antioxidants in Fresh-Cut Carrot through a Synergistic Effect with Wounding

Bernadeth B. Surjadinata [1], Daniel A. Jacobo-Velázquez [2] and Luis Cisneros-Zevallos [2,*]

[1] Department of Horticultural Sciences, Texas A&M University, College Station, TX 77843-2133, United States; Bernadeth_Surjadinata@bayvalleyfoods.com (B. B. Surjadinata), lcisnero@tamu.edu (L.Cisneros-Zevallos)

[2] Tecnológico de Monterrey, Escuela de Ingeniería y Ciencias, Centro de Biotecnología FEMSA, Av. Eugenio Garza Sada 2501 Sur, C.P. 64849 Monterrey, N.L., México; djacobov@itesm.mx (D. Jacobo-Velazquez)

* Correspondence: lcisnero@tamu.edu; Tel.: +001-979-845-3244

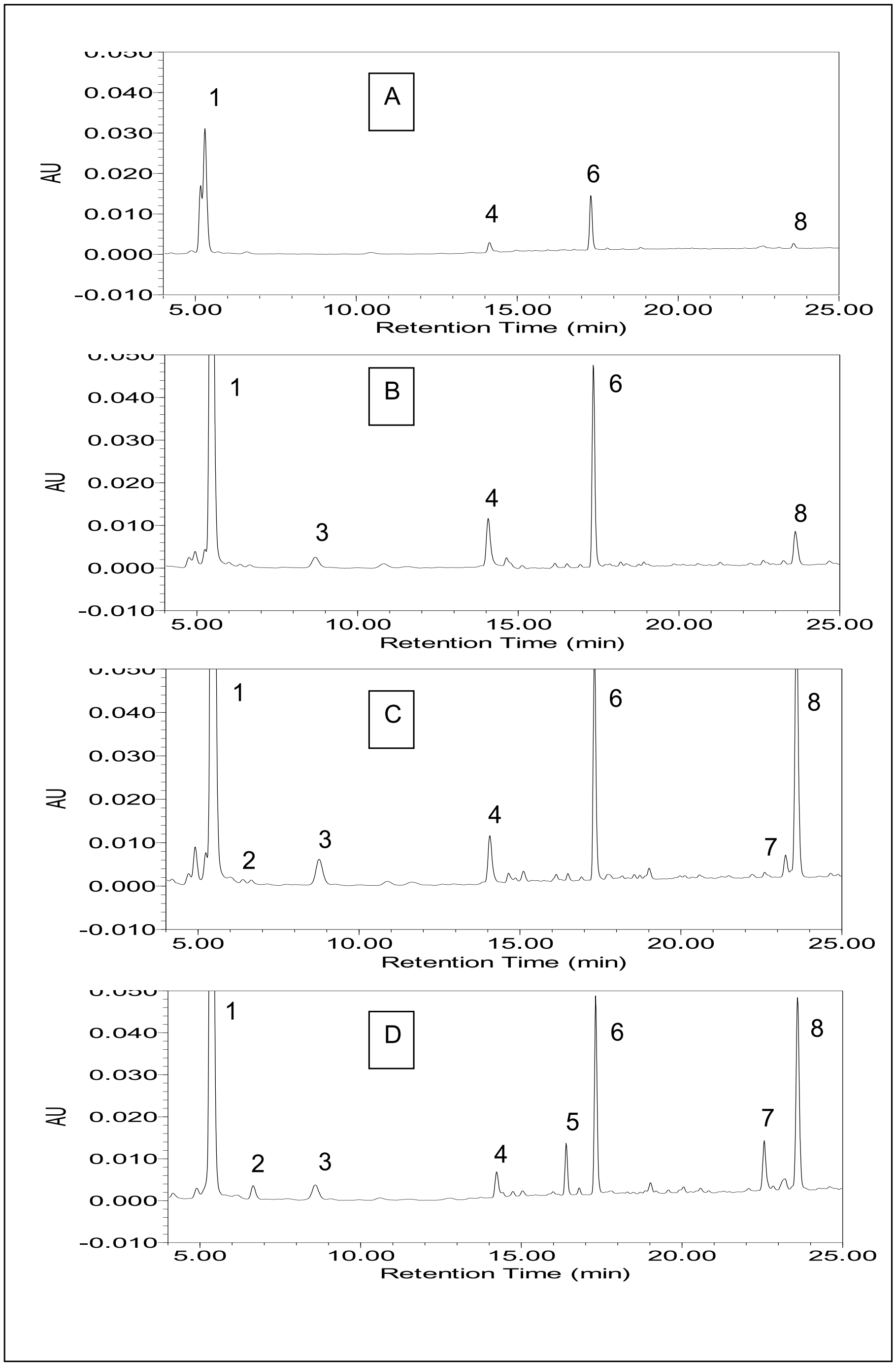

Figure S1. HPLC phenolic profiles of non-wounded carrots (A), slices (B), pies (C), and shreds (D) at 280 nm after 15 min of exposure to UVC (11.8 W/m^2) and 4 d storage at 15°C. Peaks: 1 = chlorogenic acid (5-CQA), 2 = *p*-hydroxybenzoic acid (*p*HBA), 3 = *p*-coumaric acid derivative, 4 = ferulic acid (FA), 5 = 3,5-dicaffeoylquinic acid (3,5-diCQA), 6 = 4,5-dicaffeoylquinic acid (4,5-diCQA), 7 = hydroxybenzoic acid (HBA) derivative, 8 = isocoumarin. AU = absorbance unit.

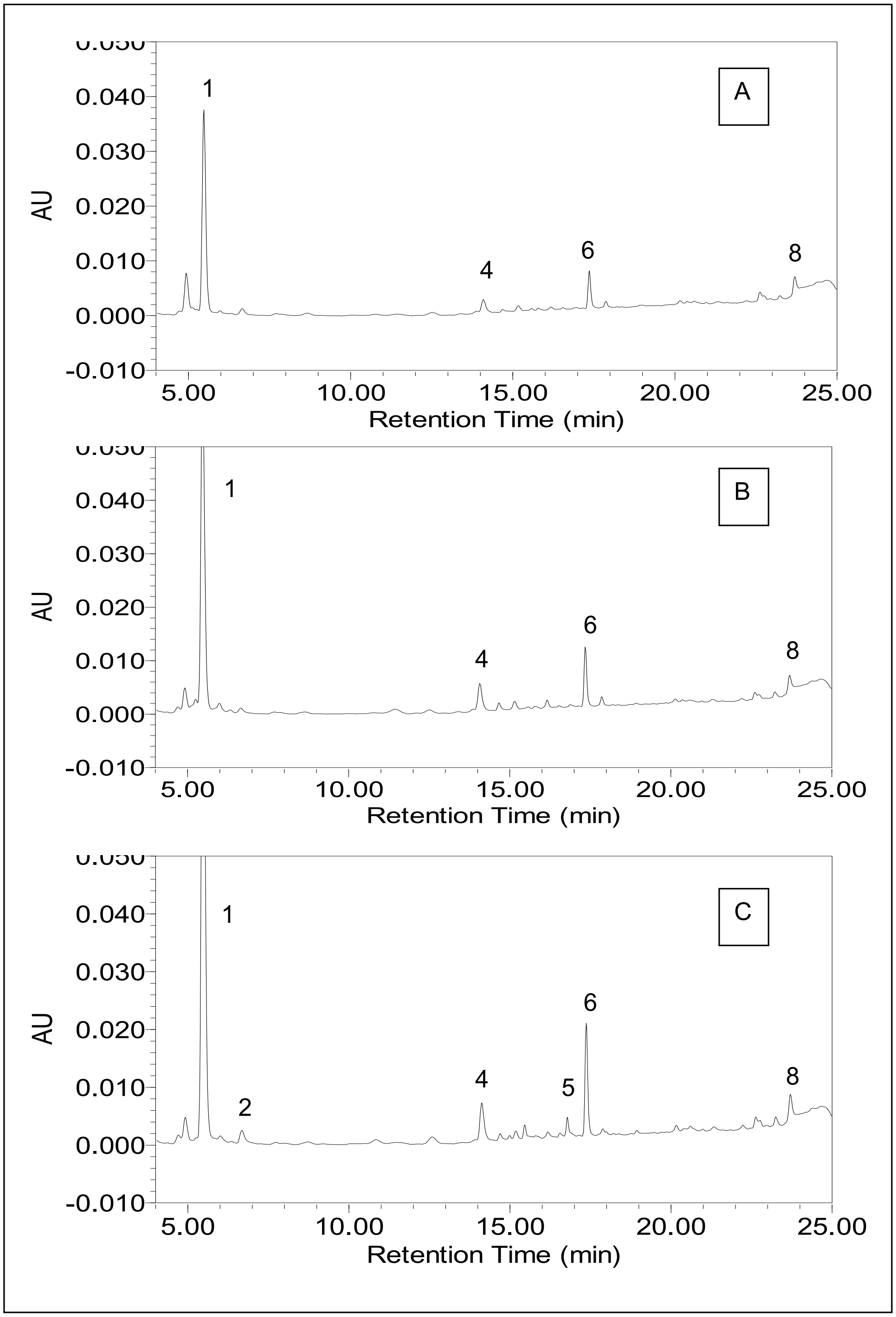

Figure S2. HPLC phenolic profiles of carrot pies at 280 nm after exposure to 0 min (A), 60 min (B), and 360 min (C) of UVA (12.7 W/m^2). Measurements were taken after 4 d storage at 15°C. Peaks: 1 = chlorogenic acid (5-CQA), 2 = *p*-hydroxybenzoic acid (*p*HBA), 3 = *p*-coumaric acid derivative, 4 = ferulic acid (FA), 5 = 3,5-dicaffeoylquinic acid (3,5-diCQA), 6 = 4,5-dicaffeoylquinic acid (4,5-diCQA), 7 = hydroxybenzoic acid (HBA) derivative, 8 = isocoumarin. AU = absorbance unit.

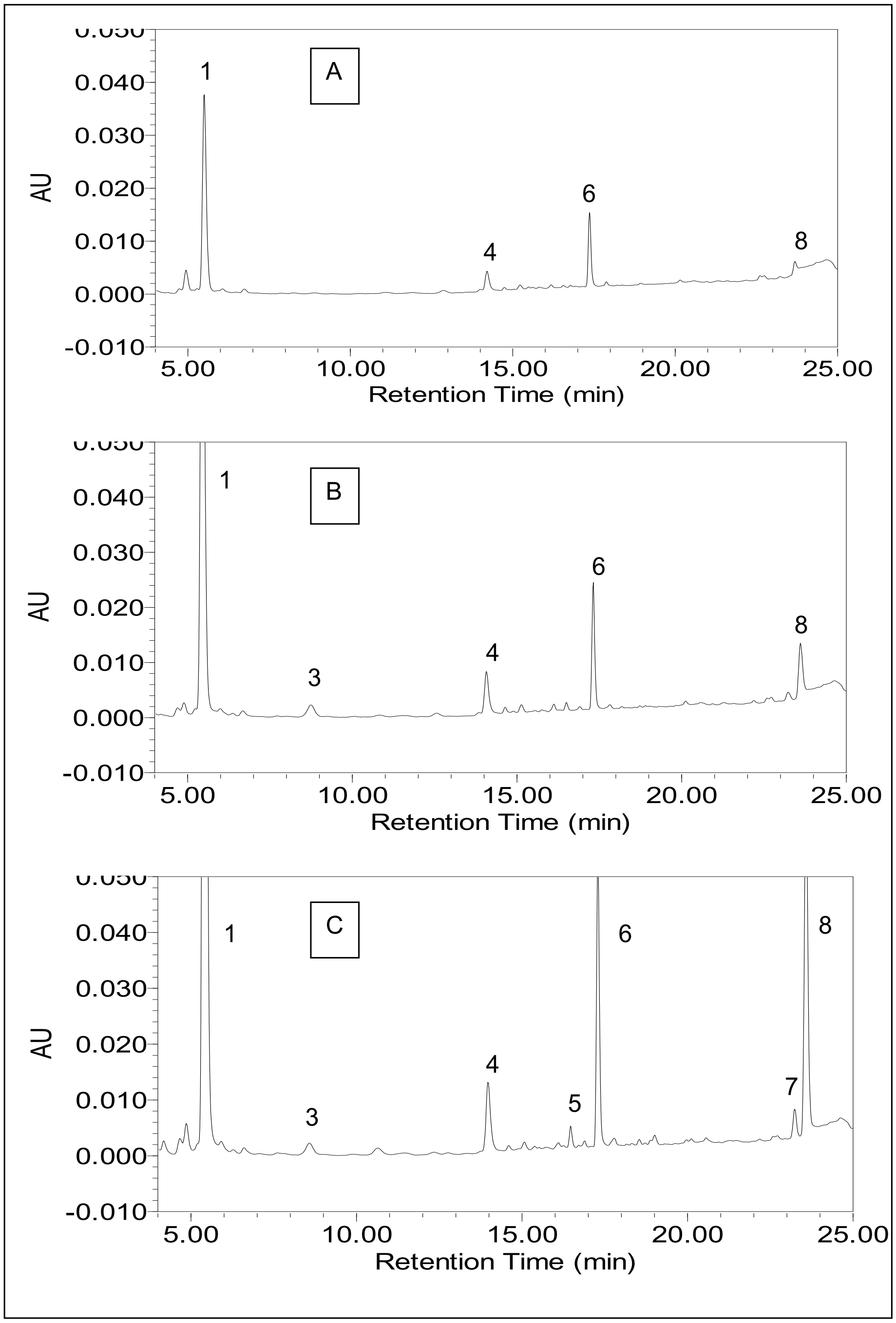

Figure S3. HPLC phenolic profiles of carrot pies at 280 nm after exposure to 0 min (A), 60 min (B), and 360 min (C) of UVB (10.4 W/m²). Measurements were taken after 4 d storage at 15°C. Peaks: 1 = chlorogenic acid (5-CQA), 2 = *p*-hydroxybenzoic acid (*p*HBA), 3 = *p*-coumaric acid derivative, 4 = ferulic acid (FA), 5 = 3,5-dicaffeoylquinic acid (3,5-diCQA), 6 = 4,5-dicaffeoylquinic acid (4,5-diCQA), 7 = hydroxybenzoic acid (HBA) derivative, 8 = isocoumarin. AU = absorbance unit.

Table S1. Individual phenolic content (mg/100g FW) of non-wounded and cut carrots exposed to UVC (11.8 W/m²). Quantification was based on HPLC profiles at 280 nm. Measurements were taken after 4 d storage at 15°C

Peak	Compound	Whole radiated with UVC (min)				Slices radiated with UVC (min)				Pies radiated with UVC (min)				Shreds radiated with UVC (min)			
		**0**	**0.5**	**1**	**15**	**0**	**0.5**	**1**	**15**	**0**	**0.5**	**1**	**15**	**0**	**0.5**	**1**	**15**
1	5-CQA	10.00	8.45	9.61	11.17	12.00	26.99	40.80	52.12	9.21	27.01	36.19	75.52	44.70	60.71	65.42	58.78
2	*p*-HBA	nd*	nd	nd	0.56	nd	nd	nd	0.35	nd	nd	nd	1.16	nd	nd	nd	1.12
3	*p*-coumaric acid derivative	nd	nd	nd	nd	nd	nd	nd	0.44	nd	nd	nd	0.86	nd	nd	nd	1.21
4	FA	1.77	1.71	1.83	1.60	1.95	2.25	2.82	3.24	1.79	2.29	2.38	2.38	2.49	2.64	2.52	2.31
5	3,5-diCQA	nd	nd	nd	nd	nd	nd	nd	nd	nd	nd	nd	nd	4.91	5.29	6.03	5.83
6	4,5-diCQA	6.64	7.26	7.41	6.36	6.61	7.74	10.77	12.02	6.37	8.13	10.19	12.45	13.23	13.57	14.00	12.27
7	HBA derivative	nd	nd	nd	2.10	nd	nd	nd	1.44	nd	nd	nd	3.44	nd	nd	nd	3.58
8	Isocoumarin	0.04	0.05	0.04	0.04	0.06	0.55	0.23	0.30	0.06	0.16	0.92	1.84	0.59	1.07	1.45	1.31

* nd = not detected

Table S2. Individual phenolic content (mg/100g) of non-wounded and carrot pies exposed to UVA (12.7 W/m[2]). Quantification was based on HPLC profiles at 280 nm. Measurements were taken after 4 d storage at 15°C.

Peak	Compound	Whole	Pie-cut radiated with UVA		
			0 min	60 min	360 min
1	5-CQA	7.73	13.98	18.55	29.67
2	*p*-HBA	nd*	nd	nd	0.37
3	*p*-coumaric acid derivative	nd	nd	nd	nd
4	FA	1.57	1.73	2.17	2.36
5	3,5-diCQA	nd	nd	nd	4.60
6	4,5-diCQA	4.93	5.35	5.74	6.85
7	HBA Derivative	nd	nd	nd	nd
8	Isocoumarin	0.05	0.11	0.10	0.12

* nd = not detected

Table S3. Individual phenolic content (mg/100g) of non-wounded and carrot pies exposed to UVB (10.4 W/m[2]). Quantification was based on HPLC profile at 280 nm. Measurements were taken after 4 d storage at 15°C.

Peak	Compound	Whole	Pie-cut radiated with UVB		
			0 min	60 min	360 min
1	5-CQA	7.82	13.15	38.84	70.63
2	*p*-HBA	nd*	nd	nd	nd
3	*p*-coumaric acid derivative	nd	nd	0.33	0.38
4	FA	1.59	1.90	2.68	3.28
5	3,5-diCQA	nd	nd	nd	4.56
6	4,5-diCQA	4.95	6.42	7.95	13.33
7	HBA derivative	nd	nd	nd	1.03
8	Isocoumarin	0.04	0.06	0.31	1.56

* nd = not detected
